# Predictive Risk Factors of Nonalcoholic Fatty Liver Disease in a Lean Chinese Population

**DOI:** 10.3390/jpm12121958

**Published:** 2022-11-26

**Authors:** Lu Liu, Xiaolan Shi, Jingwen Gao, Chunfang Xu, Xiaolin Liu

**Affiliations:** Department of Gastroenterology, The First Affiliated Hospital of Soochow University, Suzhou 215000, China

**Keywords:** non-alcoholic fatty liver disease, lean population, predictive model, nomogram

## Abstract

Background: Although nonalcoholic fatty liver disease (NAFLD) is related to obesity, it may also affect lean individuals. Recent data suggest that lean NAFLD patients can develop the whole spectrum of NASH. However, the NAFLD predictive model for lean populations remains lacking. Methods: A total of 5037 lean individuals were included in this study, and the data were separated for training and validation. The logistic regression method was used, and a nomogram, a type of prediction model, was constructed according to the logistic regression analysis and the significant clinical factors. The performance of this model was evaluated based on its discrimination, calibration, and clinical utility. Results: The individuals were divided into the training (*n* = 4068) or validation (*n* = 969) cohorts at a ratio of 8 to 2. The overall prevalence of NAFLD in the lean cohort was 6.43%. The nomogram was constructed based on seven predictors: alanine aminotransferase, total cholesterol, triglycerides, low-density lipoprotein cholesterol, creatinine, uric acid, and hemoglobin A1C. The model based on these factors showed good predictive accuracy in the training set and in the internal validation set, with areas under the curve (AUCs) of 0.870 and 0.887, respectively. The calibration curves and decision curve analysis (DCA) displayed good clinical utility. Conclusion: the nomogram model provides a simple and reliable ability to predict the risk of NAFLD in lean subjects. The model can predict lean NAFLD and can help physicians screen and identify lean subjects at a high risk of NAFLD.

## 1. Introduction

Nonalcoholic fatty liver disease (NAFLD) is the most common liver disease in industrialized countries and is an emerging issue in East Asia. NAFLD is characterized by the accumulation of fat in more than 5% of the hepatocyte and is not associated with alcohol consumption [1]. NAFLD is a disease that covers a wide spectrum, ranging from simple steatosis to nonalcoholic steatohepatitis (NASH), NASH-related liver fibrosis, cirrhosis, and hepatocellular carcinoma [2,3,4,5]. NAFLD is indirectly associated with an increase in metabolic syndrome, which also includes abdominal obesity, insulin resistance, elevated blood pressure, altered fasting glucose, and dyslipidemia [6,7]. In addition, NAFLD has been found to be closely related to many extrahepatic comorbidities such as colorectal adenoma, cardiovascular disease, type 2 diabetes mellitus, chronic kidney disease, and neurological system diseases.

While the prevalence of NAFLD is higher in subjects with obesity, NAFLD can also be found in individuals who are not obese or are lean, which is commonly the case in Asians [8]. The subset of NAFLD individuals with BMI < 25 kg/m^2^ is termed “non-obese NAFLD” [9], but the definition of “lean NAFLD” varies with different BMI cut-off points among races. In Asian populations, lean NAFLD is often used to refer to patients whose BMI is below 23 kg/m^2^ [10,11]. Some studies demonstrated that one fifth of all NAFLD patients are individuals who are not obese. Recently, an epidemiological study reported that the prevalence of non-obese or lean NAFLD increased dramatically among East Asian populations. A Taiwanese study showed a prevalence of 12.7% for NAFLD in individuals who are non-obese [12]. Additionally, a Korean study revealed a prevalence of 23.4% in non-obese adults during a health checkup [13].

Epidemiological studies on NAFLD have been conducted in populations of people with obesity; however, much less is known about NAFLD in individuals who are lean. Despite lean subjects with NAFLD having milder metabolic abnormalities (e.g., dyslipidemia, insulin resistance, hypertension, and diabetes), they can develop the whole spectrum of NASH including steatosis, lobular inflammation, hepatocyte ballooning, and/or fibrosis [14]. To improve the management and prevention of this disease, obtaining a useful predictive tool for lean populations is important to realize the factors associated with lean NAFLD. Nomograms have been widely used in developing predictive models of disease. This study aims to construct and validate a nomogram to detect NAFLD in the lean Chinese population.

## 2. Method

### 2.1. Study Population

A cross-sectional study was conducted on adults who presented NAFLD during a health checkup at the First Affiliated Hospital of Soochow University from January 2022 to June 2022, and the protocol was approved by the Ethics Committee of the First Affiliated Hospital of Soochow University (approval number: 2022-232).

**Figure 1:**
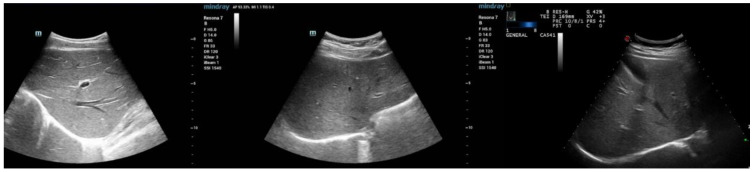
The left abdominal image shows a normal liver without steatosis. The middle image shows mild steatosis in a liver. The right image shows moderate–severe steatosis in a liver.

Patients with NAFLD were diagnosed based on the presence of hepatic steatosis in their abdominal ultrasonography findings, confirmed by multiple professional radiologists. For a diagnosis of steatosis to be made from the ultrasounds, criteria such as the detection of liver brightness; contrast between the liver and the kidney; and the appearance of the liver parenchyma, intrahepatic vessels, and diaphragm had to be met. Steatosis of a fatty liver can be categorized as mild steatosis (fat content over 5%) or moderate–severe (fat content over 20–30%) (Figure 1) [15]. The population in this study included both mild and moderate–severe steatosis groups based on their abdominal ultrasound but excluded cirrhosis and secondary causes of fatty liver diseases, especially chronic liver disease (such as viral hepatitis or autoimmune hepatitis) or alcohol consumption (>210 g/week in men and >140 g/week in women) [16]. In addition, subjects who did not have data relating to abdominal imaging findings or had a body mass index (BMI) of ≥23 kg/m^2^ were excluded from the study.

### 2.2. Clinical Assessment

**Figure 2:**
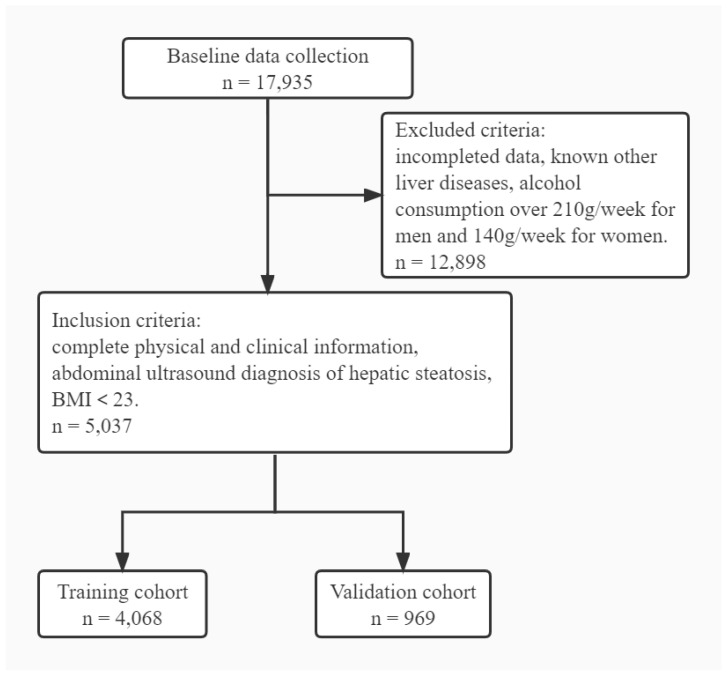
Flowchart of the study. A total of 17,935 individuals were initially collected at baseline, and 12,898 individuals were excluded due to incomplete data. The remaining 5037 subjects were randomly assigned to the training cohort (*n* = 4068) or validation cohort (*n* = 969) at a ratio of 8:2.

Clinical data including blood pressure, sex, and date of birth were recorded. The blood samples used to assess for biochemical function and used in the blood routine tests were collected after fasting for 8 h. Biomedical parameters including fasting serum glucose, albumin, total bilirubin, direct bilirubin, indirect bilirubin, gamma-glutamyltransferase (GGT), alkaline phosphatase alanine (ALP), aminotransferase (ALT), aspartate transaminase (AST), total proteins, total cholesterol, triglycerides, high-density lipoprotein (HDL), low-density lipoprotein (LDL), creatinine, urea, urea acid (UA), and hemoglobin A1C (HbA1c) were obtained. Hematological indicators including white blood cell (WBC), red blood cell (RBC), platelet, lymphocytes, monocytes, and neutrophils were measured. In total, 28 routine clinical and laboratory parameters were collected. The flowchart of the study is shown in Figure 2.

### 2.3. Statistical Analysis

Continuous data were presented as means with standard deviations, while categorical variables were shown as frequencies with percentages. A univariate logistic regression analysis combined with a multivariate logistic regression analysis (backward elimination) was performed to select the optimal predictive factors. The univariate and multivariate analyses were explored to identify the predictive variables strongly associated with lean NAFLD. The features were presented as odds ratio (OR) and 95% confidence intervals (95% CI). A value of *p* < 0.05 was considered statistically significant. All statistical analyses and graphic plotting were performed in R software (version 4.1.0, R Foundation for Statistical Computing, Vienna, Austria) (http://www.R-project.org (accessed on 20 November 2022).

### 2.4. Establishment and Evaluation of the Nomogram

To build the nomogram, the dataset was divided into training and validation sets randomly at a ratio of 8:2 and the variables were compared. A model predicting NAFLD in a lean population was constructed according to the logistic regression analysis, and the significant clinical factors were used to construct the nomogram. The performance of the nomogram was assessed in terms of calibration and discrimination, identified using a calibration curve and a receiver operating characteristic (ROC) curve, respectively [17,18]. 

## 3. Results

### 3.1. Demographic and Clinical Characteristics of Patients

The overall characteristics of patients are presented in Table 1. The overall prevalence of NAFLD in the lean cohort was 6.43%. Additionally, 252 and 72 individuals developing NAFLD were included in the training and validation sets, respectively. The total number of patients, 5037, were randomly split into the training and validation cohorts at a ratio of 8 to 2 (*n* = 4068 in the training set and *n* = 969 in the validation set). Of the subjects in the NAFLD group, 42.3% were male, with a median age of 48 years. Patients with NAFLD were typically older and had higher values in the lipid and renal metabolic panels.

### 3.2. Selection of Predictive Factors 

A univariate logistic regression analysis identified 26 variables as potential risk factors: age, sex, WBC, RBC, platelet, lymphocytes, monocytes, neutrophils, systolic blood pressure (SBP), diastolic blood pressure (DBP), glucose, HbA1c, urea, creatinine, UA, total protein, albumin, indirect bilirubin, GGT, ALP, total cholesterol, triglycerides, HDL, LDL, ALT, and AST. Factors that significantly affected lean NAFLD in the univariate analysis were included in a multivariate analysis, which demonstrated that sex, age, RBC, platelet, ALT, total cholesterol, triglycerides, LDL, creatinine, UA, and HbA1c were independent predictive factors associated with the presence of NAFLD in lean individuals (Table 2). The features were presented as OR and 95% CI, and a *p* value < 0.05 was considered statistically significant.

### 3.3. Development of Nomogram

Eleven variables were selected in the multivariate logistic regression analysis: age, gender, RBC, platelet, ALT, total cholesterol, triglycerides, LDL, creatinine, UA, and HbA1c. To develop a simple-to-use nomogram, the predictive variables were selected according to the logistic regression analysis findings and clinical application. Compared with other factors, age, sex, RBC, and platelet carried less weight in the overall model and obtained small scores on the risk score panel for its respective categories. Consequently, the nomogram was constructed by incorporating nine predictors, including ALT, total cholesterol, triglycerides, LDL, creatinine, UA, and HbA1c. Each factor was scored using the top points scale, and the overall points were added to the lowest total points scale to obtain the NAFLD risk for the evaluated individual.

### 3.4. Evaluations of the Nomogram Performance

**Figure 3:**
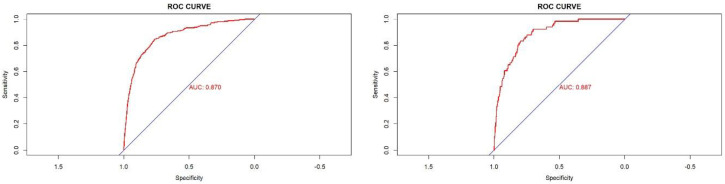
ROC curve of the predictive model and in the training cohort (**left**) and validation cohort (**right**).

**Figure 4:**
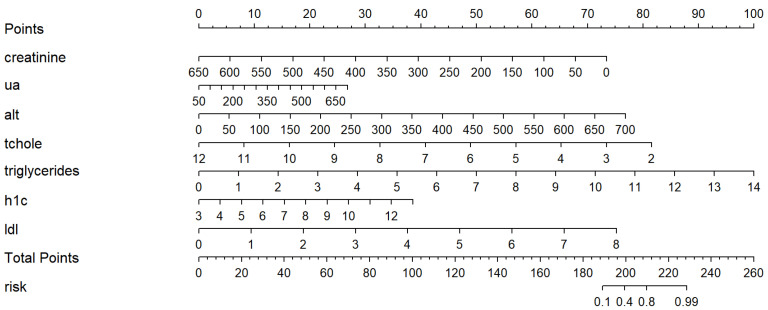
Nomogram for predicting NAFLD in the lean Chinese population. Each variable for the individuals was scored by the top points scale, and then, the points for each variable were added. Finally, personalized risk of NAFLD was obtained according to the bottom total points scale. NAFLD, nonalcoholic fatty liver disease; ua, uric acid; alt, alanine aminotransferase; tchole, total cholesterol; h1c, hemoglobin A1c; ldl, low-density lipoprotein cholesterol.

**Figure 5:**
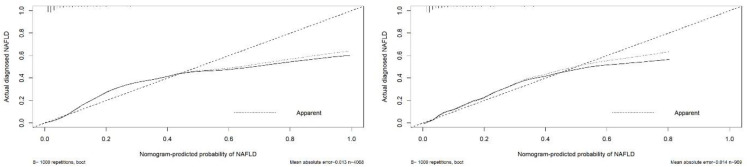
Calibration curve of the predictive model in the training cohort (**left**) and validation cohort (**right**). The X-axis represents the predicted risk of NAFLD in a lean population. The Y-axis represents the actual occurrence rate of NAFLD in a lean population.

**Figure 6:**
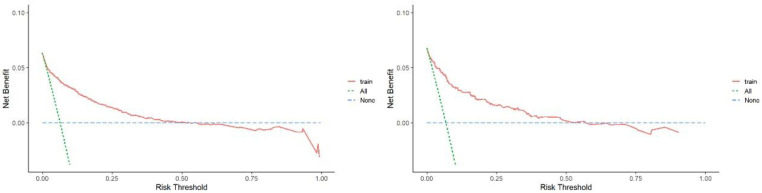
DCA of the predictive model in the training cohort (**left**) and validation cohort (**right**). The green line represents the net benefit if all patients receive treatment. The red line represents a net benefit of 0 if all patients do not receive treatment. The red solid curve above the green and blue lines represents the added net benefit if the model is used compared with either the treat-all or treat-none strategies.

Evaluations of the nomogram’s validity were conducted on its discrimination, calibration, and clinical utility by plotting an ROC curve, a calibration curve, and a DCA curve. As is shown in Figure 3, in the training cohort, the area under the curve (AUC) of the nomogram was 0.870. In the internal validation cohort, the AUC was 0.887. These results indicate that the nomogram was efficient in distinguishing between subjects with NAFLD and non-NAFLD in a lean population (Figure 4). The calibration of the prediction model was evaluated, and a calibration curve was obtained (Figure 5). The decision curve analysis showed good clinical value and a wide range of benefits (Figure 6).

## 4. Discussion

In recent decades, with the changes in lifestyle and diet in China, the burden of NAFLD has increased. Previous models predicted that China will see the greatest growth in NAFLD prevalence compared with the rest of the world in 2030 [19]. Although NAFLD is strongly observed in individuals with overweight or obesity, type 2 diabetes, or metabolic syndromes, almost 20% of patients with NAFLD are estimated to have lean or nonobese body habitus [20]. Clinically, most NAFLD in lean patients is diagnosed incidentally during imaging examinations for other medical illnesses [21]. Developing a simple and practical predictive tool is critical for screening individuals in the lean population who have a potential risk of NAFLD, especially in routine annual physical examinations when abdominal ultrasounds are not ordered. In this study, a convenient and practical nomogram was developed and validated to detect NAFLD in lean subjects. Seven parameters were included in the nomogram: ALT, total cholesterol, triglycerides, LDL, creatinine, UA, and HbA1c. These parameters make the nomogram an objective and easy-to-use predictive tool for screening patients for lean NAFLD and may help contribute to a better clinical diagnosis and early prevention.

Dyslipidemia is a well-known metabolic feature in NAFLD. Our study found that increased levels of cholesterol, triglycerides, and LDL are risk factors in lean NAFLD. Experimental and clinical evidence suggested that an increase in intrahepatic cholesterol might be related to NAFLD progression and that the accumulation of cholesterol in the liver was a possible pathological mechanism of NASH development [22,23,24]. In the current study, dyslipidemia, especially hypertriglyceridemia is a vital factor for NAFLD development, progression and regression and is associated with NAFLD in lean subjects [12,25]. Hypertriglyceridemia and hepato-steatosis are generally believed to result from an increase in FFA. Studies have reported that lean NAFLD subjects had more visceral adiposity, which leads to a high level of FFA in the liver and exacerbates hepatic triglyceride accumulation [26]. During the pathophysiological process of hyperglyceridemia, the peripheral adipose tissue increase lipolysis and the hepatocytes increase the uptake of FFA [27]. In addition, hypertriglyceridemia can stimulate de novo lipogenesis and decrease FFA oxidation in the liver, which have significant influence on hepatic steatosis [28]. In line with our study results, previous studies reported that hyperglyceridemia is an independent parameter contributing to the development of NAFLD in lean individuals [11]. Khalid’s team from Saudi Arabia revealed that cholesterol and LDL were correlated with lean fatty liver in patients as well, especially among female individuals [29]. ALT is an indicator of liver injury with high sensitivity and specificity, and studies revealed that elevated ALT was a manifestation of NAFLD. Wang et al. found that ALT was demonstrated to be an independent risk factor in NAFLD and was even higher among female subjects in the lean group compared with those in the overweight/obese groups [30,31]. Conversely, not all the NASH patients had elevated ALT, and one fourth of patients with NAFLD have a normal value of ALT [32,33].

The results indicated that HbA1c was markedly increased by the presence of NAFLD in lean subjects and, thus, is a risk factor, and this increase provides a possible pathological link with metabolic irregularities and insulin resistance. The current study revealed that insulin resistance plays a vital role in the pathogenesis of NAFLD in subjects who are lean and in those who are obese, regardless of metabolic syndrome. The possible mechanism of insulin resistance in NAFLD is that increased hepatic insulin and impaired glucose are involved in hepatic de novo lipogenesis, which activate sterol regulatory element-binding protein 1c (SREBP-1c) and carbohydrate response element-binding protein (ChREBP), respectively. These processes contribute to hepatic free fatty acids (FFA) accumulation, which plays a major role in NAFLD pathogenesis [34,35,36,37,38,39].

Moreover, UA and creatinine are significant risk factors in the lean NAFLD group. A disturbance in UA has been reported to possibly be responsible for an impairment in lipid metabolism and insulin resistance through the NLRP3 inflammasome. In the liver, UA was regulated by the oxidative stress process in mitochondria, and a high level of UA in hepatocytes might lead to an enhancement of lipid superoxide, which exacerbates hepatic steatosis [40,41]. The reduction in creatinine associated with NAFLD might be due to sarcopenia, which means low skeletal muscle mass and reduced function. Studies have reported that skeletal muscle mass was lower in lean patients with NAFLD, compared with that in subjects who are obese. Recent studies found that insulin resistance, mitochondrial dysfunction, and chronic low-grade inflammation were recognized as important causative factors for sarcopenia in NAFLD [42,43,44,45,46].

In the study, we developed a nomogram using a large sample to diagnose the general lean population for NAFLD, and the model showed good discrimination, calibration, and clinical utility. However, the potential limitations of our study should also be mentioned. First, patients with NAFLD were diagnosed based on hepatic ultrasound examination, which does not provide a severity score without a biopsy examination and omits the liver fat content. Second, some clinical variables such as waist circumference and lifestyle information were not included, resulting in a possible selection bias. Third, we did not collect patients’ medication information; some medications could affect liver function, such as station, which might protect the liver from steatosis [47]. Lastly, this nomogram was constructed with single-center data and was not validated by an external hospital. Thus, multicentric investigations are still needed. 

In conclusion, in this study, we constructed a nomogram based on seven predictors—ALT, total cholesterol, triglycerides, LDL, creatinine, UA, and HbA1c—associated with NAFLD in lean individuals. This nomogram can be deployed as a simple tool that would help physicians in screening and identifying lean subjects at high risk of NAFLD.

## Figures and Tables

**Table 1:** The clinical characteristics of the study population.

Variables		Training Dataset (*n* = 4068)			Validation Dataset (*n* = 969)		
	Group	Non-NAFLD	NAFLD	*p*-Value	Non-NAFLD	NAFLD	*p*-Value
Sex (%)	Male	2714 (71.2)	104 (40.3)	<0.001	632 (70.0)	33 (50.0)	<0.001
	Female	1096 (28.8)	154 (59.7)		271 (30.0)	33 (50.0)	
Age (Years) (mean (SD))		40.00 (13.00)	48.00 (13.00)	<0.001	40.00 (12.00)	48.00 (13.00)	<0.001
SBP (mmHg) (mean (SD))		119.00 (16.00)	128.00 (19.00)	<0.001	119.00 (16.00)	126.00 (14.00)	<0.001
DBP (mmHg) (mean (SD))		72.00 (10.00)	77.00 (10.00)	<0.001	72.00 (10.00)	77.00 (8.00)	<0.001
Urea (mmol/L) (mean (SD))		4.80 (1.31)	5.01 (1.19)	0.012	4.79 (1.24)	4.88 (1.11)	<0.001
Cr (mmol/L) (mean (SD))		59.17 (17.05)	63.68 (14.08)	<0.001	59.05 (20.90)	62.00 (12.03)	<0.001
UA (mmol/L) (mean (SD))		300.40 (71.74)	366.35 (76.41)	<0.001	299.82 (71.51)	347.69 (72.24)	<0.001
TP (g/L) (mean (SD))		72.26 (3.57)	73.48 (3.64)	<0.001	72.40 (3.55)	73.68 (3.56)	0.003
Albumin (g/L) (mean (SD))		45.16 (2.39)	45.55 (2.59)	0.012	45.26 (2.46)	45.59 (2.25)	0.279
TB (μmol/L) (mean (SD))		15.17 (5.76)	15.08 (5.28)	0.811	15.11 (5.86)	14.10 (5.26)	0.151
DiB (μmol/L) (mean (SD))		4.26 (1.72)	4.15 (1.60)	0.296	4.24 (1.77)	3.75 (1.38)	0.02
IndiB (μmol/L) (mean (SD))		10.90 (4.22)	10.93 (3.90)	0.921	10.87 (4.33)	10.35 (4.03)	0.32
ALT (U/L) (mean (SD))		14.53 (9.58)	26.10 (20.39)	<0.001	15.24 (23.46)	24.13 (12.06)	0.001
AST (U/L) (mean (SD))		19.17 (6.12)	22.62 (8.58)	<0.001	19.49 (13.06)	21.28 (5.59)	0.247
GGT (U/L) (mean (SD))		16.46 (17.28)	35.15 (52.91)	<0.001	17.45 (34.62)	34.63 (24.76)	<0.001
ALP (U/L) (mean (SD))		56.87 (18.01)	69.51 (19.55)	<0.001	57.07 (19.94)	68.12 (16.70)	<0.001
TCh (mmol/L) (mean (SD))		4.75 (0.84)	5.01 (0.88)	<0.001	4.77 (0.87)	5.03 (0.89)	0.017
TG (mmol/L) (mean (SD))		1.03 (0.57)	1.91 (1.28)	<0.001	1.04 (0.54)	1.93 (0.88)	<0.001
HDL (mmol/L) (mean (SD))		1.52 (0.35)	1.23 (0.28)	<0.001	1.50 (0.35)	1.24 (0.26)	<0.001
LDL (mmol/L) (mean (SD))		2.74 (0.76)	3.08 (0.81)	<0.001	2.78 (0.78)	3.10 (0.82)	0.001
WBC (×10^9^/L) (mean (SD))		5.72 (1.37)	6.36 (1.50)	<0.001	5.73 (1.38)	6.53 (1.27)	<0.001
RBC (×10^9^/L) (mean (SD))		4.57 (0.44)	4.86 (0.46)	<0.001	4.56 (0.44)	4.93 (0.45)	<0.001
PLT (×10^9^/L) (mean (SD))		231.55 (55.65)	241.08 (61.24)	0.009	233.15 (53.29)	240.47 (61.14)	0.262
L (×10^9^/L) (mean (SD))		1.98 (0.54)	2.12 (0.63)	<0.001	2.00 (0.56)	2.22 (0.53)	0.002
M (×10^9^/L) (mean (SD))		0.38 (0.12)	0.43 (0.13)	<0.001	0.38 (0.12)	0.43 (0.13)	0.002
N (×10^9^/L) (mean (SD))		3.20 (1.04)	3.62 (1.14)	<0.001	3.19 (1.06)	3.69 (0.97)	<0.001
glucose (mmol/L) (mean (SD))		5.04 (0.69)	5.74 (1.66)	<0.001	5.04 (0.68)	5.62 (1.32)	<0.001
HbA1C (mg/dL) (mean (SD))		5.58 (0.47)	6.04 (0.91)	<0.001	5.58 (0.48)	5.99 (0.67)	<0.001

ALP, alkaline phosphatase; DBP, diastolic blood pressure; DiB, direct bilirubin; GGT, gamma-glutamyl transferase; HbA1C, hemoglobin HbA1c; HDL, high-density lipoprotein; IndiB, indirect bilirublin; L, lymphocyte; LDL, low-density lipoprotein; M, monocytes; N, neutrophil; SBP, systolic blood pressure; TB, total bilirublin; TCh, total cholesterol; TG, total triglycerides; TP, total protein.

**Table 2:** The results of the logistic regression analysis for NAFLD in lean individuals.

Variables	Univariate Analysis		Multivariate Analysis	
	OR [95% CI]	*p* Value	OR [95% CI]	*p* Value
Age (Years)	1.037 [1.029, 1.044]	<0.001	1.039 [1.025, 1.052]	0.000
Sex (male vs. female)	3.341 [2.659, 4.208]	<0.001		
SBP (mmHg)	1.028 [1.022, 1.034]	<0.001	0.999 [.0987, 1.011]	0.852
DBP (mmHg)	1.050 [1.038, 1.060]	<0.001	1.013 [.0995, 1.031]	0.164
Urea (mmol/L)	1.097 [1.017, 1.178]	<0.001	0.911 [0.807, 1.029]	0.134
Cr (mmol/L)	1.006 [1.002, 1.011]	<0.001	0.975 [0.962, 0.988]	0.000
UA (mmol/L)	1.010 [1.008, 1.011]	<0.001	1.007 [1.004, 1.009]	0.000
TP (g/L)	1.099 [1.065, 1.134]	<0.001	1.025 [0.980, 1.073]	0.281
Albumin (g/L)	1.067 [1.018, 1,118]	<0.001	1.049 [0.977, 1.127]	0.187
DiB (μmol/L)	0.929 [0.864, 0.996]	<0.001	0.994 [0.907, 1.089]	0.902
ALT (U/L)	1.037 [1.030, 1.045]	<0.001	1.056 [1.038, 1.074]	0.000
AST (U/L)	1.032 [1.018, 1.046]	<0.001	0.923 [0.895, 0.951]	0.000
GGT (U/L)	1.019 [1.015, 1.023]	<0.001	1.003 [0.997, 1.009]	0.341
ALP (U/L)	1.025 [1.020, 1.030]	<0.001	1.004 [0.998, 1.011]	0.214
TCh (mmol/L)	1.376 [1.219, 1.549]	<0.001	0.309 [0.175, 0.545]	0.000
TG (mmol/L)	3.413 [2.963, 3.946]	<0.001	2.670 [2.027, 3.516]	0.000
HDL (mmol/L)	0.043 [0.028, 0.068]	<0.001	0.774 [0.344, 1.739]	0.535
LDL (mmol/L)	1.632 [1.435, 1.854]	<0.001	3.484 [2.051, 5.918]	0.000
WBC (×10^9^/L)	1.357 [1.263, 1.458]	<0.001	0.005 [0.000, 16.971]	0.199
RBC (×10^9^/L)	4.108 [3.242, 5.214]	<0.001	1.821 [1.163, 2.849]	0.009
PLT (×10^9^/L)	1.003 [1.001, 1.004]	<0.001	1.004 [1.002, 1.007]	0.000
L (×10^9^/L)	1.579 [1.316, 1.891]	<0.001	23.829 [0.062, 92.046]	0.194
M (×10^9^/L)	14.358 [6.467, 41.533]	<0.001	12.613 [0.028, 56.740]	0.260
N (×10^9^/L)	1.396 [1.273, 1.527]	<0.001	23.973 [0.063, 91,755]	0.193
glucose (mmol/L)	1.786 [1.611, 1.986]	<0.001	1.067 [0.890, 1.279]	0.484
HbA1C (mg/dL)	2.477 [2.110, 2.927]	<0.001	1.428 [1.096, 1.861]	0.008
TB (μmol/L)	0.991 [0.970, 1.010]	0.373	-	-
IndiB (μmol/L)	0.995 [0.967, 1.021]	0.700	-	-

ALP, alkaline phosphatase; DBP, diastolic blood pressure; DiB, direct bilirubin; GGT, gamma-glutamyl transferase; HbA1C, hemoglobin HbA1c; HDL, high-density lipoprotein; IndiB, indirect bilirublin; L, lymphocyte; LDL, low-density lipoprotein; M, monocytes; N, neutrophil; SBP, systolic blood pressure; TB, total bilirublin; TCh, total cholesterol; TG, total triglycerides; TP, total protein.

## Data Availability

Not applicable.
